# From lemming to leader: Moving beyond Gross Domestic Product (GDP) to bring health financing assistance into the 21^st^ century

**DOI:** 10.1371/journal.pgph.0003135

**Published:** 2024-05-01

**Authors:** Tiffany Nassiri-Ansari's, Nina Schwalbe, Susanna Lehtimaki

**Affiliations:** 1 Spark Street Advisors, New York, NY, United States of America; 2 United Nations University–International Institute for Global Health, Kuala Lumpur, Malaysia; 3 Mailman School of Public Health, Columbia University, New York, NY, United States of America; PLOS: Public Library of Science, UNITED STATES; McGill University, CANADA

Nearly 90 years after Simon Kuznets first introduced Gross Domestic Product (GDP) for the limited purpose of measuring economic growth (by measuring the monetary value of all local goods and services within a given period of time), calls continue to mount for decision-makers to stop using GDP and its derivate, Gross National Income (GNI), for purposes far beyond their original design [1]. This is particularly true in the case of the development assistance architecture, where these indicators are used as proxies to measure a nation’s overall well-being and, in some cases, eligibility for external funding [2, 3]. The GNI-based classification system has recently even been suggested by some Member States as a criterion to access to medicines in the new WHO Pandemic Agreement.

In recent years, increasing scrutiny of “transition” processes for countries shifting from low- to middle-income classification status has revealed that the use of these measures to determine eligibility for and allocation of aid consistently prioritizes economic growth over the health of populations [4]. Yet while recognizing GDP and GNI are not fit for purpose, major health financing agencies, including Gavi, the Vaccine Alliance, the Global Fund to Fight Aids, Tuberculosis and Malaria, and the World Bank, continue to use these measures to determine eligibility [2, 3, 5]. They do this in part because of ease of calculation, overall generalizability, availability of data to calculate these indicators annually and methodological simplicity and consistency across countries [6].

As Gavi, among other funds, heads towards a replenishment event this spring, it may be time to call it quits on the use of these measures to allocate global health aid.

Why? Because GDP- and GNI-based criteria mask inequalities within and between countries [6]. As aggregate figures measuring solely economic activity [7], GDP and GNI render invisible disparities caused by gender, race, ethnicity, religion, (dis)ability, geography, socioeconomic status, and more, particularly disparities exacerbated by intersecting axes of marginalization. They also exclude a significant proportion of those in poor health living under the poverty line. Middle-income countries (MICs) collectively host 70% of the world’s poor and an increasing proportion of the global burden of disease, yet are often ineligible for aid since MICs are assumed to have ‘graduated’ from being eligible for aid regardless of potential gaps between health expenditure and needs [8–10]. This mismatch between economic growth and population health drives home the fact that measures of economic activity cannot be used to assess the strength of health systems and the health of the populations they serve. This status quo must be disrupted well ahead of the 2030 deadline for the Sustainable Development Goals (SDGs), as the attainment of SDG 3, Good Health and Wellbeing, hinges upon the provision of universal health coverage (UHC), amongst other targets. It has been estimated that attainment of health-related SDG targets “will cost LICs and MICs an additional US$371 billion per year in health spending by 2030,” driving home the reality that SDG 3 is only achievable if sufficient health aid is allocated to the vast majority of the world’s poor, who are residents of MICs [9].

The good news is, there are viable options for change. These include “add-on” measures, such as on sustainability and green growth, or “alternatives” replacing GDP and GNI altogether; options from both categories have been implemented in the real world at different levels (often sub-national or nation-wide) and to varying degrees of success [6].

Two particularly promising alternatives stand out. One is Aotearoa New Zealand’s Living Standards Framework (LSF) [11], which incorporates a holistic understanding of wellbeing for national budgeting that is locally relevant and adjustable according to government priorities. The customizable and flexible nature of this measure allows for the identification of diverse and nuanced health needs, capacities, and challenges. The other is the Oxford Poverty and Human Development Initiative’s Multidimensional Poverty Index (MPI) [12] which includes a customizable dashboard approach distinguishing it from the “one-size-fits-all” models of GDP and GNI. As indicated by its name, the MPI allows for a multi-dimensional calculation and understanding of poverty that is capable of countering the ‘masking’ effect GDP and GNI have on inequities and disparities.

Such alternatives could be adopted at scale, with a few basic adjustments. First, calculating them requires reliable, available, and timely data; adoption is thus contingent upon selection of and agreement on a shared set of indicators which can be collected and reported in a manner that is time-, energy-, and cost-efficient [13]. Second, they must fulfill prerequisites on standardization, applicability, and acceptability; the feasibility and utility of these alternatives must be clearly identified and articulated as value-adds to socialize a new approach amongst governments, policymakers, and the public at large. And finally, donors must shift to a paradigm that delinks progress from the “growth at any cost” mindset [14] and recognizes that economic growth is not necessarily correlated with health system performance nor the improvement of population health.

In 2022, Joseph Stiglitz asserted that “GDP should be dethroned”–while also warning that another one-size-fits-all measure should not take its place [15]. Indeed, there are viable options for international financing agencies to move from GDP- and GNI-based criteria to more nuanced, contextualized, and customizable measures designed to assess the needs of populations and health systems around the world, towards the financing and attainment of good health and wellbeing for all by 2030 [6]. As demonstrated by the real-world implementation of the LSF, MPI, and other alternatives, a shift away from GDP and GNI towards more robust measures is possible; however, this urgent shift can only be made a reality through strong political will generated through a coalition of global health agencies, country-level ministries (such as health, finance, and economy), and other relevant actors.

Most crucially, however, this shift must be situated within and serve to advance a broader effort to reform global health aid architecture, dispelling the myth of aid as charity for low-income countries to reframe it as a collective funding pool to facilitate the attainment of good health and wellbeing for everyone, everywhere.
