# Correction: From lemming to leader: Moving beyond Gross Domestic Product (GDP) to bring health financing assistance into the 21^st^ century

**DOI:** 10.1371/journal.pgph.0004789

**Published:** 2025-06-10

**Authors:** Tiffany Nassiri-Ansari, Nina Schwalbe, Susanna Lehtimaki

The first author’s name is spelled incorrectly. The correct name is Tiffany Nassiri-Ansari. The correct citation is: Nassiri-Ansari T, Schwalbe N, Lehtimaki S (2024) From lemming to leader: Moving beyond Gross Domestic Product (GDP) to bring health financing assistance into the 21st century. PLOS Glob Public Health 4(5): e0003135. https://doi.org/10.1371/journal.pgph.0003135.
